# A behavioral health needs assessment and general psychological well‐being of digital and multimedia forensic examiners

**DOI:** 10.1111/1556-4029.70207

**Published:** 2025-10-31

**Authors:** Sonali Tyagi, Kathryn C. Seigfried‐Spellar

**Affiliations:** ^1^ Department of Computer & Information Technology Purdue University West Lafayette Indiana USA

**Keywords:** CSAM, digital forensics examiner, mental health, perceived need for care, psychological well‐being, PTSD, vicarious trauma

## Abstract

Research shows that digital forensic examiners experience high stress levels due to the nature of their jobs involving exposure to disturbing media. This study conducted a needs analysis by examining digital and multimedia forensic examiners' psychological well‐being, coping mechanisms, social support, and attitudes toward and experiences with barriers to counseling and mental health support. Ninety‐four digital and multimedia forensic examiners (DFE) completed the anonymous online survey. Respondents were also asked to self‐report their primary duty within digital forensics (e.g., image, audio, or video analysts) and whether they were also working as an investigator/detective; 53 were DFE‐only, and 41 had dual roles (DFE + detective). Results examined differences in primary duties (e.g., image vs. non‐image analyst) and the number of primary duties (e.g., two vs. three). Of the sample, 36% personally sought counseling due to work‐related stress. Image forensic analysts reported more psychological distress and barriers toward help‐seeking compared with audio and video analysts. 17% (*n* = 16) of the sample met the diagnostic criteria for PTSD. There were no significant differences between DFE‐only and those working dual roles as detectives on psychological well‐being and attitudes toward mental health support. Finally, digital forensic examiners who met the diagnostic criteria for PTSD reported 9 out of 15 mental health stigmas, many of which included fear associated with agency culture (e.g., “affect my promotion”). Findings support the need for accessible, agency‐supported, and potentially mandated mental health services for DFE to improve well‐being and resiliency.

**Highlights**

- Image forensic examiners experienced more psychological symptoms.
- Image forensic examiners scored higher on the PTSD scale.
- Counseling was required for only a few participants.
- Digital forensic examiners reported several mental health stigmas and barriers to help‐seeking.

## 1 INTRODUCTION

Digital evidence is estimated to be a factor in about 90% of criminal cases [1], with mobile phones being the most seized device in criminal investigations [2]. Digital evidence is defined as information that is either transferred or stored via a computer [3], which may be found on mobile phones, laptops/tablets/computers, global positioning systems (GPS) devices, gaming systems, surveillance cameras, Internet of things (IoT) devices, and networks, to name a few. The advancement of digital technology has changed the criminal investigation process; due to the proliferation of digital devices, we now have images and videos of the actual crime itself, not just eyewitness accounts to help investigators narrate the story [4, 5]. According to Bayly [6], “video [recorded on smartphones] is turning social media into a virtual crime scene.”

In digital forensics, while evidence is routinely collected from a multitude of devices, the unique complexities of image, audio, and video files necessitate highly specialized training for digital forensic examiners to ensure accurate analysis and interpretation [7, 8]. For instance, digital forensic examiners are often requested to enhance audio/video/image evidence to identify the actors or clarify the context of the situation, all of which may involve repeatedly viewing or hearing heinous acts (e.g., homicide, child sexual exploitation, and abuse, torture) [4, 5]. Researchers agree that digital forensic examiners are at a heightened risk for work‐related stress [5, 9, 10], but limited research exists on their perceived need for mental health care. According to Villatoro et al. [11], the perceived need for care is “a person's awareness that something is wrong, that what is wrong relates to their mental health, and that professional assistance is necessary to overcome it” (p. 2). Previous research on perceived need for care focuses on the military [12, 13, 14] and the general population [15, 16]. For the current study, we aimed to understand DFE' perceived need for mental health care by examining their psychological well‐being and attitudes toward and experiences with barriers to counseling and mental health support.

## 2 LITERATURE REVIEW

Research shows that digital forensic examiners are exposed to high levels of stress due to the sheer volume of digital evidence [5, 9, 17, 18, 19, 20]. In such occupations, during the investigation and review, examiners are exposed to potentially distressing material [5, 10, 18, 21, 22] of challenging and traumatic events, including violence [5, 23], suicide [5, 23], death scenes [5], child sexual abuse material [5, 10, 17, 24], and sexual assault [5, 17]. Moreover, the complexity of digital evidence, due to increased storage capacity [20], the number of devices acquired during the investigation [25], and the backlog of cases [5, 22], contributes to workplace stress [5].

Digital forensic examiners and investigators dealing with disturbing media may experience a tremendous amount of emotional burnout [19, 22], anxiety [5], emotional stress [9], physical illness [9, 18], poor psychological well‐being [5, 18, 22], and vicarious trauma (i.e., secondary traumatic stress; [10, 18, 22]). Digital forensic examiners and investigators also often exhibit poor psychological well‐being outside their work environment [9, 17, 18, 19].

Research suggests that the viewing medium for disturbing content may impact digital forensic examiners differently. For instance, if the reviewing medium is in the form of a video, digital forensic examiners experience more psychological symptoms compared with other digital mediums [17]; however, Perez et al. [19] found that viewing images related to child abuse was the most challenging part for investigators and digital forensic examiners. Seigfried‐Spellar et al. [4] analyzed the differences in reported stress by multimedia forensic examiners for various traumatic events (e.g., rape, suicide, police shooting, child abuse) based on the type of evidence (image, video, or audio). At least 90% of the multimedia forensic analysts found audio, video, and image digital evidence of child abuse to be stressful, the highest of the different traumatic events assessed. The second most stressful event for all types of digital evidence was rape [4].

Moreover, large organizations may have full‐time digital forensic examiners; however, in small organizations, traditional policing and forensic examiner roles may overlap, creating more job stress for examiners who must split their time between multiple roles [10, 18, 19]. As per the study conducted by Seigfried‐Spellar [10], digital forensic examiners who also work as investigators scored higher on PTSD as compared with individuals with either a digital forensic examiner role or an investigator‐only role. However, a study by Seigfried‐Spellar et al. [4] on multimedia forensic examiners (audio, video, and image analysts) found the opposite in that multimedia forensic examiners with a joint detective/investigator role reported higher job satisfaction, positive affect, and overall psychological well‐being compared with those who worked solely as a multimedia forensic examiner. In addition, Holt and Blevins [18] suggest that individuals with fewer conflicting roles within an agency report higher levels of job satisfaction and moderate levels of work‐related stress.

Other contributing factors to the increasing risk of stress for law enforcement professionals, including investigators and forensic examiners, are organizational stress (e.g., administrative and supervisory roles; [9, 18]), occupational stress (e.g., high demands and unrealistic expectations; [5, 26]), and personal stress (work‐life conflict; [19]). Work‐related stress may impact either job performance, focused attention, or decision‐making capabilities [27]. Moreover, technical limitations at work, such as frequent software crashes and slow processing times also contribute to burnout among digital forensic examiners in the workplace environment [28]. In addition, increased work‐related stress relates to low job satisfaction [9, 29], and individuals may be more aggressive and negative toward fellow colleagues [9, 30, 31] and their intimate partners [5].

Research suggests that continuous viewing of distressing media and excessive workload may impact the psychological well‐being of an individual, resulting in post‐traumatic stress disorder (PTSD), which is also known as secondary traumatic stress (STS) [17]. In addition, if digital forensic examiners view disturbing content for an extended period, then they have a high risk of having PTSD symptoms [5]. For example, Perez et al. [19] found that 18% of the participants were diagnosed with high levels of STS, and 18% were diagnosed with moderate levels because of reviewing disturbing media over a long period of time. Seigfried‐Spellar et al. [4] found that 28% of digital forensic examiners met the diagnostic criteria for STS. Moreover, PTSD symptoms may have a direct association with dissatisfaction in intimate partner relations [32]. Law enforcement personnel dealing with child sexual abuse material (CSAM) face challenges related to relationship intimacy [24, 33, 34] and reduced desire for physical connection with their partners [35]. A study conducted by Denk‐Florea et al. [17] also revealed that digital forensic examiners may have trouble maintaining relationship intimacy with their partners if they review distressing media during their work.

Coping skills are essential in helping individuals manage stressful events and decide how they respond to critical or major workplace incidents [24, 26]. Digital forensic examiners use various coping mechanisms to reduce stress, including dark humor [5, 17], adaptive strategies (e.g., outside activities; [10, 18]), and maladaptive strategies (e.g., substance abuse; [10, 22]). Maladaptive strategies may reduce stress in the short term but increase stress in the long term [5] and decrease work productivity [26]. Strong social support is an important coping mechanism for reducing stress and improving psychological well‐being [17, 26]. For instance, digital forensic examiners reported lower levels of PTSD and better psychological well‐being if their close ones supported their work [19, 22].

Although an adaptive coping mechanism, there is a stigma associated with seeking mental health support [36, 37, 38]. Many digital forensic examiners may be reluctant to receive mental health services [10, 17], and when they do, it is primarily to deal with PTSD symptoms [22]. In addition, few agencies support mandatory counseling/treatment for law enforcement professionals exposed to distressing content [10, 19, 22], especially for digital forensic examiners working on child exploitation cases [10]. There are multiple reasons for mental health stigma, including a lack of information about mental health services, a negative perception of mental illness, and a fear of facing discrimination after receiving a mental health diagnosis [36]. Thus, building an organizational culture to support mental well‐being and normalizing mental illness is essential to reducing mental health stigma [17].

## 3 CURRENT STUDY

Digital forensic examiners play a crucial role in examining digital evidence to support law enforcement investigations. Although they may collect digital evidence from a variety of devices (e.g., smartphones, laptops), the key media types (e.g., images, audio, and video) require specific tools and expertise to be interpreted correctly [3]. For example, image forensics involves metadata extraction, sensor pattern noise analysis, and tamper detection [39, 40, 41], all of which require enhancing the visibility of details in an image without altering it [42]. Audio forensics encompasses authenticating audio, enhancing its clarity, identifying speakers, and interpreting content, regardless of its origin (e.g., surveillance systems, mobile devices, or intercepted communications; [43, 44, 45]). Finally, video forensic examiners perform frame‐by‐frame analysis, scene reconstruction, and motion tracking [42, 46, 47] while also adjusting visual parameters to improve visibility [48].

Thus, the current study aimed to understand DFE' perceived need for care based on their primary duty as image, video, and audio analysts. DFE completed an anonymous survey assessing psychological well‐being, coping mechanisms, social support, and attitudes toward and experiences with barriers to counseling and mental health support. Based on previous research, we expected to find differences in psychological well‐being, PTSD, job satisfaction, and coping mechanisms between DFE based on their primary duties. In addition, we expected to find that DFE experience barriers to counseling and mental health support due to workplace stigma. However, with the increased awareness about the mental health challenges facing digital forensic examiners, we hoped to find more positive attitudes toward and experiences with mental health support than previously cited in the literature.

## 4 METHODS

### 4.1 Participants

Raw scores included 190 participants; however, 13 respondents were deleted as they were suspected of being bots or multiple submission attempts according to Qualtrics' fraudulent score parameters. About 31 participants were deleted due to incomplete data, as they consented to the survey, but then immediately dropped out. A total of 52 respondents consented to the survey but were disqualified (i.e., 35 were not certified digital forensic examiners; 17 were not permanent US residents). The final sample for statistical analyses included 94 certified digital forensic examiners.

As given in Table 1, most respondents were Caucasian/White (*n* = 85, 90.4%), male (*n* = 71, 75.5%), and married (*n* = 66, 70.2%); ages ranged from 24–68 years old (*M* = 43.10, *SD* = 8.82); and years working as DFE ranged from 1–4.5 years (*M* = 8.96, *SD* = 6.24). The respondents self‐reported their duties as digital forensic examiner‐only (i.e., DFE) or digital forensic examiner and detective/investigator (DFE + DET). Fifty‐three participants worked solely as digital forensic examiners, and 41 worked both as digital forensic examiners and detectives/investigators.

**TABLE 1:** Sample demographics.

Variable		DFE (*n* = 53)	DFE + DET (*n* = 41)	Total (*N* = 94)
Gender	Male	35 (66.0)	36 (87.8)	71 (75.5)
	Female	18 (34.0)	5 (12.2)	23 (24.5)
Age	18–24	2 (3.8)	0 (0.0)	2 (2.1)
	25–34	7 (13.2)	7 (17.1)	14 (14.9)
	35–44	19 (35.8)	18 (43.9)	37 (39.4)
	45–54	21 (39.6)	13 (31.7)	34 (36.2)
	55 or older	4 (7.5)	3 (7.3)	7 (7.4)
Ethnicity	Caucasian/White	45 (84.9)	40 (97.6)	85 (90.4)
	Others	8 (15.1)	1 (2.4)	9 (9.6)
Marital Status	Single	15 (28.3)	5 (12.2)	20 (21.3)
	Married, CL, CU	35 (66.0)	31 (75.6)	66 (70.2)
	D, S, W	3 (5.7)	3 (7.3)	6 (6.4)
	Missing	0 (0.0)	2 (4.9)	2 (2.1)
Agency	City	21 (39.6)	25 (61.0)	46 (48.9)
	County	9 (17.0)	13 (31.7)	22 (23.4)
	State	11 (20.8)	2 (4.9)	13 (13.8)
	Federal	3 (5.7)	0 (0.0)	3 (3.2)
	NGO/Private	7 (13.2)	1 (2.4)	8 (8.5)
	Other	2 (3.8)	0 (0.0)	2 (2.1)
Rank	Sworn Officer	9 (17.0)	36 (87.8)	45 (47.9)
	Civilian	35 (66.0)	4 (9.8)	39 (41.5)
	Other	9 (17.0)	1 (2.4)	10 (10.6)
Years Working as DFE	<5	13 (24.5)	16 (39)	29 (30.9)
	5–9	12 (22.6)	14 (34.1)	26 (27.7)
	10–14	15 (28.3)	5 (12.2)	20 (21.3)
	14–19	5 (9.4)	4 (9.8)	9 (9.6)
	≥20	8 (15.1)	0 (0.0)	8 (8.5)
	Missing	0 (0.0)	2 (4.9)	2 (2.1)

*Note*: *N* = 94. Note any discrepancies in percentage due to rounding. Values represent frequencies with percentages in parentheses.

Abbreviations: CL, Common Law; CU, Civil Union; D, Divorced; DFE, Digital Forensics Examiner‐Only; DFE + DET, Detective/Investigator who also works as Digital Forensic Examiners who are also Detectives; S, Separated; W, Widowed.

### 4.2 Measures

Participants completed an anonymous online survey assessing demographics, use of coping mechanisms, job satisfaction, and a behavioral health needs assessment (BHNAS). The BHNAS was adapted from the Naval Unit Behavioral Health Needs Assessment Survey (NUBHNAS; [13]) and included several questionnaires measuring psychological well‐being, social support, and attitudes toward and experiences with mental health care (i.e., barriers to mental health). This study was approved by the authors' Institutional Review Board (IRB # 2020–1452).

### 4.3 Psychological health and well‐being

The NUBHNAS measured psychological distress using the K10 and K6 item pool [49]. K10 and K6 measured psychological symptoms such as depressed mood (*α* = 0.91), anhedonia (*α* = 0.75), eating habit changes (*α* = 0.20), sleep pattern changes (*α* = 0.44), motor agitation (*α* = 0.85), motor retardation (*α* = 0.75), fatigue (*α* = 0.71), worthless guilt (*α* = 0.90), concentration (*α* = 0.79), death/suicide thoughts (*α* = 0.57), anxiety (*α* = 0.87), worry (α = 0.77), motor tension (*α* = 0.76), hypersensitivity (*α* = 0.86), vigilance (*α* = 0.90), and positive affect (*α* = 0.86). All Cronbach's alphas were acceptable, except for changes in eating habits and sleep patterns– these variables should be considered with caution.

The PTSD Checklist for DSM‐5 (PCL‐5; [50]) version assessed the possibility of STS. The PCL‐5 comprises 20 items corresponding to the symptomatic responses to STS and is a standardized self‐administered reporting scale. The example items include questions such as “Feeling very upset when something reminds you of a stressful case?” or “Suddenly acting or feeling as if a stressful case was happening again (as if you were reliving it)?” The items were on a 5‐point Likert scale (0 = Not at all; 4 = Extremely). The total symptom severity score of 20 items ranges from 0 to 80, with any score above 31 considered a possibility of PTSD [50]. To confirm that the participants indicated symptoms of work‐related stress arising from analyzing disturbing media, we included the following statement at the start of the scale: “As a forensic analyst/technician, you work cases involving digital evidence that can be extremely stressful. Because of your role as a forensic analyst, how often in the past month have you experienced…” Cronbach's alpha for PCL‐5 was *α* = 0.96, an acceptable value.

### 4.4 Job satisfaction

One question measured job satisfaction on a scale of 1 (extremely dissatisfied) to 5 (extremely satisfied), adapted from [51] Quality of Employment Survey: “All in all, how satisfied are you with your job?”

### 4.5 Coping mechanisms

The coping mechanisms used by participants to deal with their stress, whether healthy or unhealthy, were assessed using a version of a 16‐item coping scale [18]. Authors have assessed 19 questions under seven categories of coping mechanisms as discussed by Holt and Blevins [18], such as “distraction or suppression” (e.g., “I work harder than usual…”) having six items (1–5 and 19); “using substances” (e.g., “I take a sedative…”) having five items (6–10); “talking with others” (e.g., “I talk things…”) having three items (11–13); “withdrawal or escapism” (e.g., “I try to be alone.”) having 1 item (14); prayer/meditation (e.g., “I engage in some religious…”) having 1 item (15); problem‐solving (e.g., “I seek professional help…”) having 1 item (16); and eating habits (e.g., “I eat more”) having two items (17–18) (see Table 5 for the item number references). These questions were measured on a scale of 1 (Never) to 6 (Always). This coping mechanism scale aims to measure the variety of coping strategies used by the participants to deal with work‐related stress while going through disturbing media.

### 4.6 Social support

The Duke Social Support Index (DSSI) measured participants' social networks and how those networks support them. The DSSI's shorter version comprises 11 items containing three subscales, which measure (1) social interaction (four items), (2) satisfaction with social support (six items), and (3) social support (10 items by combining social interaction items and social support satisfaction items) [52]. In this study, the author used a 6‐item scale to measure digital forensic examiners' satisfaction with social support. They were asked questions such as, “Does it seem that your family and friends (people who are important to you) understand you?” or “Can you talk about your deepest problems with at least some of your family and friends?” etc. The questions were measured on a scale of 1 (Hardly), 2 (Some of the time), and 3 (All of the time). The participants were also given an option to “Decline to Respond.” Cronbach's alpha (*α* = 0.86) has an acceptable value, which shows good reliability. For scoring, the average was calculated for six items of “satisfaction with social support.”

### 4.7 Behavioral health needs assessment

We examined the participants' beliefs in whether therapy would improve their symptoms via the Adapted Credibility/Expectancy Questionnaire (CEQ; [53]). Respondents used a sliding scale (0 to 100%) to rate their beliefs toward the following three questions: (1) “By the end of the therapy, how much improvement in your symptoms do you think would occur;” (2) “How much do you really feel that therapy will help you reduce your symptoms;” and (3) “How much improvement in your symptoms do you really feel would occur.”

Respondents completed 14 items (1 = Strongly Disagree; 5 = Strongly Agree) from the Mental Health Advisory Team (MHAT) survey, which assessed the barriers and stigma associated with mental health care [13]. These items considered the factors that may impact decisions to receive mental health counseling, such as “I don't trust mental health professionals” or “It is difficult to get an appointment.” A 15th item was added to the MHAT survey: “It is difficult to talk about the things I have seen.” Cronbach's alpha was acceptable at 0.92 for the 15‐item survey, indicating good reliability.

Furthermore, participants answered eight questions assessing their attitudes toward counseling/treatment [10]: “The following questions ask about your experience with counseling or treatment as a result of your work‐related stress as a digital forensic examiner, multimedia forensic analyst, and/or investigator/detective.” They reported “Yes,” “No,” or “Indifferent” to questions such as: “Have you ever sought counseling/treatment as a result of your work‐related stress,” and “Do you personally know someone who has sought counseling/treatment as a result of work‐related stress.”

### 4.8 Procedure

Members of the digital and multimedia forensics community were solicited via several listservs, such as the National White Collar Crime Center (NW3C), Law Enforcement Video Association (LEVA), and Internet Crimes Against Children (ICAC) task force. Due to the sensitive nature of this study, a self‐reported internet‐based survey was completely anonymous– no identifying information was collected to reduce bias and increase the validity of self‐report responses on socially sensitive topics [54]. The anonymous internet‐based survey was hosted on Qualtrics, and participants were encouraged to forward the recruitment email to other DFE. Participants were required to self‐report that they were at least 18 years of age, a permanent resident of the USA, *and* currently working as a certified digital or multimedia forensic examiner to participate in the study. DFE who completed the online survey were redirected to a separate “Thank You” page, where they could enter their email address to be compensated for their participation with a $10.00 Amazon e‐gift card.

### 4.9 Analytical strategies

We used IBM SPSS Statistics V29 for all statistical analyses and set the alpha level at 0.10. Marginal significance was considered (*p* < 0.10) as we wanted to reduce the risk of Type II error [55]; we determined the risk of not diagnosing someone with potential mental health issues (i.e., suicidal) was greater than misdiagnosing someone. In addition, we emphasized effect sizes as they are a better indication of the magnitude of the relationship than *p*‐values, which are a function of sample size [55, 56]. For the current study, we interpreted correlations as an effect size, with clinical importance approaching 0.20 [57, 58], ω^2^ for ANOVA with 0.01 (small), 0.06 (medium), and 0.14 (large; [59]), and *r* for *t*‐test [55].

Based on their self‐reported job role, we created the variable: digital forensic examiner‐only (DFE‐Only) vs. digital forensic examiner and detective/investigator (DFE + DET). In addition, based on their self‐reported duties, several dichotomous variables were created: Image Analyst vs. Non‐image Analyst; Audio Analyst vs. Non‐Audio Analyst; and Video Analyst vs. Non‐Video Analyst. Due to individuals having overlapping roles or duties, we also created a grouping variable that explored the potential impact of working as either forensic analysts or detectives: one primary role, two primary roles, three primary roles, all four primary roles (i.e., image, audio, video, and detective), and “other.” These individuals who reported their primary job duties as “other” wrote in responses, such as computer forensics, mobile forensics, or drone forensics.

Additionally, a dichotomous variable for PTSD (No/Yes) indicated which individuals met the DSM‐5 diagnostic criteria for PTSD based on the scoring guidelines (i.e., scores ≥31 suggested PTSD) provided by Weathers et al. [50]. Finally, items related to seeking or receiving mental health counseling were dichotomous (No, Yes). All other variables of interest (e.g., general psychological well‐being, PTSD sum, job satisfaction, coping mechanisms, social support, mental health attitudes) were continuous.

First, we examined the relationship between the dichotomous primary duties (e.g., Image vs. Nonimage Analyst) and the number of years working as a digital forensic examiner for general psychological well‐being (e.g., Depression), job satisfaction, coping mechanisms, mental health stigma, and social support using zero‐order correlations. Means and standard deviations were also calculated for all continuous variables, and one‐way ANOVAs with post hoc tests (i.e., Bonferroni, Gabriel) explored mean differences between the number of job duties (One vs. All Four) and PTSD (No/Yes). Chi‐square tests also examined the relationship between counseling, PTSD (No/Yes), and the various primary duties. The overall goal of these analyses was to better understand the perceived needs for mental health support and general psychological well‐being for DFE working with disturbing media.

It should be noted that in our initial analyses, we did not control for the number of years working as a digital forensic examiner; however, partial correlations controlling for the number of years working as a digital forensic examiner can be found in supplementary materials (see next section). In general, we found few differences in psychological well‐being and mental health attitudes after controlling for the number of years working as a DFE.

## 5 RESULTS

### 5.1 Descriptives

Regarding primary duty on the job, participants were more likely to report multiple primary duties rather than working solely as an image, audio, or video forensic technician/examiner. For instance, 6 (6.4%) individuals reported working as a detective *and* all three primary digital forensic duties (i.e., image, audio, and video). In addition, 28 (29.8%) participants reported working all three primary digital forensic duties (image, audio, and video). Only 19 individuals reported one primary digital forensic duty as either an image (*n* = 6) or video analyst (*n* = 13). Six (6.4%) participants reported their primary duty as “other.” No participant reported working solely as an audio forensic examiner. Due to the overlapping duties and small cell counts, authors could not compare individuals who only worked as image, audio, or video forensic analysts. Thus, we created dichotomous variables and coded respondents as to whether they reported that duty as primary (No/Yes). 50 (53.2%) participants reported their primary duty as an image analyst, 27 (28.7%) as audio, and 56 (59.6%) as a video forensic examiner.

Of the 94 examiners, 16 (17%) met the diagnostic criteria for PTSD (scores ≥31): 9 (56.3%) digital forensic examiners‐only and 7 (43.8%) digital forensic examiners and detectives. When examining the number of primary roles for the 16 participants, 7 (44%) reported only one primary role, 3 (19%) reported two primary roles, 4 (25%) reported three primary roles, and 2 (13%) respondents worked all four primary roles (i.e., image, video, audio, and detective).

Of the 94 respondents, 66 (70%) reported knowing someone who has sought counseling or treatment, with over one‐third of respondents (*n* = 34, 36.2%) reporting that they sought counseling due to work‐related stress. A total of 19% (*n* = 18) of examiners reported that counseling was required in their job position; when asked if they would attend counseling if it was offered but not required, 53% reported “Yes.” When asked if they believe counseling should be required, 50% (*n* = 47) reported “Yes”— but 23% (*n* = 22) were “Indifferent,” and 27% (*n* = 25) said “No.” Responses were nearly split between “No,” “Yes,” and “Indifferent” for “Do you think counseling would help improve productivity at work?” Finally, 38% (*n* = 36) compared with 43% (*n* = 40) of individuals thought they currently needed counseling due to work‐related stress.

Furthermore, of the 94 participants, 31% (*n* = 29) of the sample reported they had been working as a digital forensic examiner for <5 years, followed by 28% (*n* = 26) who had been working for 5–9 years, and 8.5% (*n* = 8) of the sample working for 20 years or more. For the intercorrelations matrix for each section below, as well as partial correlations controlling for the number of years working as a digital forensic examiner, supplementary data to this article can be found online at https://osf.io/df7et/overview.

### 5.2 General psychological well‐being and PTSD

As given in Table 2, there were statistically significant differences in psychological well‐being between image, audio, and video forensic examiners. Image analysts reported higher scores on 11 out of the 16 symptoms: depression, anhedonia, changes in eating habits, changes in sleeping, fatigue, concentration, hypersensitivity, and vigilance compared with non‐image analysts; and marginally significant scores on worthless guilt, anxiety, and motor tension. Audio analysts reported higher scores on three out of 16 symptoms: anhedonia, fatigue, and hypersensitivity compared with non‐audio analysts, with a marginally significant score on changes in eating habits. Finally, video analysts reported higher scores on three out of 16 symptoms: anhedonia, death, and hypersensitivity; lower scores on overall positive affect compared with non‐video analysts; and marginally significant scores on depression and worry. However, there were no statistically significant differences between digital forensic examiners and individuals working as detectives and digital forensic examiners. Additionally, there were marginal differences for the number of years an individual worked as a digital forensic examiner for two symptoms: motor retardation and vigilance.

**TABLE 2:** Zero‐order correlations between primary duties and variables of interest.

	Image	Audio	Video	DFE vs dual	DFE Years^a^
Depression	0.25*	0.17	0.20^†^	0.02	0.11
Anhedonia	0.21*	0.24*	0.23*	0.03	0.06
Eating Habits	0.22*	0.18^†^	0.09	−0.09	0.14
Sleep Change	0.27**	0.11	0.15	−0.12	0.05
Motor Agitation	0.11	0.14	0.07	−0.15	0.02
Motor Retardation	0.14	0.13	0.14	−0.04	0.18^†^
Fatigue	0.21*	0.21*	0.16	−0.08	0.02
Worthless Guilt	0.18^†^	0.08	0.17	−0.00	−0.03
Concentration	0.28**	0.15	0.09	0.05	0.02
Death	0.17	0.04	0.23*	−0.03	−0.02
Anxiety	0.18^†^	0.11	0.16	−0.09	0.04
Worry	0.16	0.17	0.19^†^	−0.07	0.03
Motor Tension	0.19^†^	0.15	0.08	−0.05	0.04
Hypersensitivity	0.29**	0.24*	0.24*	−0.01	0.02
Vigilance	0.29**	0.17	0.15	−0.06	0.20^†^
Positive Affect	−0.09	−0.12	−0.25*	0.04	−0.07
PTSD Sum	0.21*	0.06	0.01	0.13	0.06
Job satisfaction	−0.07	0.05	−0.08	−0.01	0.03
Social Support	−0.21*	−0.15	−0.14	−0.08	−0.05

*Note*: Listwise *N* = 93; DFE vs. Dual = DFE‐only vs. DFE + Detective, DFE Years = years working as a DFE; ^†^ *p* < 0.10, *p < 0.05, **p < 0.01, ***p < 0.001.

^a^ *N* = 91.

For PTSD, image forensic examiners scored higher compared with non‐image analysts (*r*_*pb*_ = 0.21, *p* = 0.05; see Table 2). There was no statistically significant relationship between PTSD scores and the other primary duties. A Chi‐square analysis was conducted to analyze the relationship between the different primary duties (e.g., non‐image vs. image) and meeting the diagnostic criteria for PTSD (No/Yes). Results suggested no statistically significant relationship between the examiners' primary duty and meeting the diagnostic criteria for PTSD. However, there was a small effect size reported for the image analysts and PTSD (Cramer's *V* = 0.14), suggesting a larger sample size might reveal clinical relevance. Finally, there was no relationship between PTSD and DFEs vs. DFE + Detective.

A one‐way ANOVA examined mean differences for PTSD scores based on the number of primary roles (excluding the “Other” group). Results suggested a marginal relationship and moderate effect size, *F*(3, 84) = 2.08, *p* = 0.109, ω^2^ = 0.04, with *post hoc* analyses revealing a mean difference between one vs. four primary roles (Bonferroni = 0.09; Gabriel = 0.05). As presented in Table 3, individuals working four primary roles scored higher on PTSD compared with individuals working one primary role. However, a 2 × 4 Chi‐square analysis between PTSD (No/Yes) and the number of primary roles (1, 2, 3, and 4) found no statistically significant relationship as 15% (*n* = 6) of individuals working one role, 23% (*n* = 3) of individuals working two roles, 14% (*n* = 4) of individuals working three roles, and 33% (*n* = 2) of individuals working all four primary roles met the diagnostic criteria for PTSD.

**TABLE 3:** Means and standard deviations for variables of interest based on number of primary roles and PTSD (no/yes).

Psychological well‐being	PTSD		Roles					Total
	No	Yes	One	Two	Three	Four	Others	
*n*	78	16	41	13	28	6	6	*N* = 94
Depression	1.78 (0.69)	2.76 (0.65)	1.78 (0.67)	2.25 (0.86)	1.98 (0.78)	2.77 (0.80)	1.57 (0.66)	1.95 (0.78)
Anhedonia	1.44 (0.63)	2.59 (0.76)	1.49 (0.65)	1.69 (0.80)	1.77 (0.83)	2.33 (1.25)	1.25 (0.27)	1.64 (0.78)
Eating habits^a^	1.66 (0.64)	2.31 (0.63)	1.62 (0.69)	1.88 (0.65)	1.89 (0.68)	2.00 (0.71)	1.75 (0.69)	1.77 (0.68)
Sleep change	1.95 (0.83)	3.09 (0.92)	1.84 (0.76)	2.31 (1.13)	2.36 (0.94)	2.58 (1.39)	2.42 (0.92)	2.14 (0.95)
Motor agitation	1.77 (0.89)	3.22 (0.84)	1.80 (0.96)	1.92 (1.02)	2.27 (1.04)	2.17 (0.61)	2.33 (1.66)	2.02 (1.03)
Motor retardation	1.42 (0.64)	2.53 (1.01)	1.40 (0.69)	1.77 (0.78)	1.70 (0.84)	1.92 (0.92)	1.92 (1.39)	1.61 (0.82)
Fatigue	2.40 (0.56)	3.22 (0.48)	2.45 (0.58)	2.54 (0.75)	2.61 (0.63)	2.97 (0.45)	2.36 (0.76)	2.54 (0.63)
Worthless guilt	1.32 (0.46)	2.52 (1.10)	1.38 (0.60)	1.79 (1.05)	1.55 (0.73)	2.04 (1.20)	1.25 (0.42)	1.52 (0.76)
Concentration	1.88 (0.79)	3.28 (0.80)	1.88 (0.81)	2.38 (1.19)	2.16 (0.86)	3.17 (0.98)	2.00 (1.00)	2.12 (0.95)
Death	1.24 (0.44)	1.81 (0.93)	1.24 (0.46)	1.58 (0.64)	1.41 (0.71)	1.42 (0.80)	1.00 (0.00)	1.34 (0.59)
Anxiety	1.69 (0.63)	2.94 (0.76)	1.73 (0.69)	2.14 (1.01)	2.01 (0.92)	2.30 (0.41)	1.67 (0.39)	1.90 (0.80)
Worry	1.76 (0.73)	2.91 (0.80)	1.85 (0.78)	2.00 (1.10)	2.05 (0.90)	2.50 (0.71)	1.50 (0.45)	1.95 (0.86)
Motor tension	1.63 (0.73)	2.88 (0.83)	1.67 (0.75)	2.00 (1.02)	1.79 (0.83)	2.92 (1.07)	1.92 (0.92)	1.85 (0.88)
Hypersensitivity	1.33 (0.40)	2.06 (0.63)	1.30 (0.37)	1.46 (0.53)	1.56 (0.61)	2.10 (0.56)	1.38 (0.40)	1.45 (0.52)
Vigilance	1.64 (0.64)	2.95 (0.95)	1.60 (0.61)	2.21 (1.07)	1.88 (0.89)	2.71 (1.02)	1.92 (0.89)	1.86 (0.85)
Positive affect	3.30 (0.84)	2.34 (0.81)	3.22 (0.92)	3.15 (0.97)	3.11 (0.91)	2.42 (0.80)	3.42 (0.58)	3.14 (0.91)
PTSD Sum	10.15 (8.39)	42.06 (9.12)	13.66 (13.37)	16.92 (16.71)	15.18 (13.95)	29.33 (19.72)	14.00 (15.21)	15.59 (14.73)
Job satisfaction	3.85 (1.23)	3.44 (1.03)	3.71 (1.25)	3.92 (0.76)	3.71 (1.44)	3.50 (0.84)	4.50 (0.55)	3.78 (1.20)
Social support	2.46 (0.46)	1.93 (0.40)	2.48 (0.42)	2.26 (0.53)	2.36 (0.51)	1.72 (0.36)	2.53 (0.45)	2.37 (0.49)

*Note*: Values represent mean scores with standard deviations in parentheses; One = DFE with only one primary role (image/audio/video/detective); Two = combination of two primary roles (e.g., image + video, image + detective, etc.); Three = combination of three primary roles; Four = all four primary roles (image + video + audio + detective); Others = digital forensic examiners who did not report one of the primary roles but a general field (e.g., mobile forensics). The psychological well‐being scale is 1 (Strongly Disagree) to 5 (Strongly Agree).

^a^ *N* = 93 due to missing data for individuals with “Three Roles.”

### 5.3 Job satisfaction

Of the 94 respondents, 71.3% (*n* = 67) reported being somewhat or extremely satisfied with their job compared with 20.2% (*n* = 19) who reported being somewhat or extremely dissatisfied. There was no statistically significant correlation between job satisfaction and primary duty or DFE vs. DFE + DET (see Table 2). See Table 3 for job satisfaction descriptives based on the number of primary roles reported and PTSD (No/Yes); there were no significant mean differences in job satisfaction between groups.

### 5.4 Coping mechanisms

As per Table 4, image forensic examiners reported coping by sleeping more than usual, taking medications such as antianxiety pills, and eating more or less than usual compared with non‐image analysts. There was a marginally significant difference in that image analysts reported coping by trying to be alone compared with non‐image analysts. Audio forensic examiners reported coping by eating less than usual compared with non‐audio analysts, and there was a marginally significant difference in that audio analysts reported coping by eating more than usual compared with non‐audio analysts. Video forensic examiners reported coping by sleeping more than usual and eating less than usual compared with non‐video analysts. There was a marginally significant difference in that video analysts reported coping by taking medication, such as antianxiety pills, compared with non‐video analysts. Both audio and video forensic analysts were less likely to find a distraction as a coping mechanism than non‐audio or non‐video analysts. Finally, individuals working as DFE + Detectives reported shopping, eating less than usual, and exercising more compared with DFE. There was a marginally significant difference in that DFE + Detectives reported sleeping more than usual compared with DFE. Additionally, there was a marginally significant difference based on the number of years working as a DFE; participants working for more years as digital forensic examiners were less likely to report shopping and taking drugs, but more likely to report taking medication as a coping mechanism.

**TABLE 4:** Zero‐order correlation between coping mechanism, DFE vs. DFE + Det, and primary duties.

	Image	Audio	Video	DFE vs dual	DFE Years^a^
Working harder	−0.04	−0.02	−0.11	0.17	0.05
Try to forget	0.00	−0.04	−0.11	0.12	0.06
Find a distraction	−0.16	−0.22*	−0.18^†^	−0.06	−0.13
Sleep more	0.33**	0.16	0.22*	−0.19^†^	0.06
Go shopping	0.06	−0.06	0.06	−0.23*	−0.19^†^
Drink alcohol	−0.02	0.01	−0.03	−0.01	−0.03
Take drugs	0.06	−0.02	−0.01	0.08	−0.20^†^
Take sedatives	0.14	−0.04	0.14	0.10	−0.09
Take medication	0.28**	0.15	0.18^†^	−0.13	0.18^†^
Smoke more	−0.05	−0.04	0.01	0.08	0.02
Talk to intimate partner	−0.10	−0.08	−0.15	−0.13	−0.05
Talk to friends	−0.11	−0.15	−0.06	−0.07	−0.06
Participate in social groups	−0.02	−0.03	−0.12	−0.10	−0.04
Try to be alone	0.20^†^	0.15	0.11	−0.02	0.14
Engage in religious activity	0.08	0.01	−0.05	−0.05	0.07
Seek professional help	0.07	0.00	0.08	−0.05	−0.06
Eat more	0.32**	0.19^†^	0.18^†^	−0.09	0.14
Eat less	0.29**	0.26*	0.23*	−0.23*	0.11
Exercise more	0.12	0.02	−0.04	−0.21*	−0.09

*Note*: Listwise *N* = 91; DFE vs. Dual = DFE‐only vs. DFE + Detective, DFE Years = years spent as a DFE; ^†^ *p* < 0.10, *p < 0.05, **p < 0.01, ***p < 0.001.

^a^ *N* = 89.

There were statistically significant mean differences between the number of primary roles and the following coping mechanisms: talking with friends, *F*(3, 84) = 2.98, *p* = 0.04, ω^2^ = 0.06; and eating more than usual, *F*(3, 84) = 3.92, *p* = 0.01, ω^2^ = 0.09. As given in Table 5, individuals with all four roles were less likely to talk to friends as compared with individuals with one role (Bonferroni = 0.04; Gabriel = 0.02) or three roles (Bonferroni = 0.03; Gabriel = 0.01). Individuals with three primary roles reported eating more as a coping mechanism compared with individuals with one primary role (Bonferroni = 0.01; Gabriel = 0.01).

**TABLE 5:** Mean and standard deviations for coping mechanisms based on number of primary roles reported and PTSD (no/yes).

Coping mech	PTSD		Roles				
	No	Yes	One	Two	Three	Four	Others
*n*	76	15	39	13	27	6	6
Working harder	3.24 (1.18)	3.67 (1.35)	3.28 (1.28)	3.08 (1.12)	3.26 (1.10)	3.67 (1.63)	3.83 (0.98)
Try to forget	3.29 (1.27)	4.13 (1.36)	3.44 (1.17)	3.23 (1.36)	3.33 (1.33)	3.83 (2.14)	3.83 (1.47)
Find a distraction	3.68 (1.24)	3.47 (1.13)	3.85 (1.27)	3.46 (1.39)	3.30 (1.17)	3.67 (0.52)	4.33 (0.82)
Sleep more	2.47 (1.29)	3.40 (1.55)	2.26 (0.99)	3.15 (1.77)	2.86 (1.41)	3.00 (1.79)	2.50 (1.76)
Go shopping	2.28 (1.18)	2.47 (1.06)	2.21 (1.13)	2.54 (1.20)	2.26 (1.13)	2.00 (0.63)	3.00 (1.79)
Drink Alcohol	2.74 (1.35)	2.87 (1.60)	2.92 (1.40)	2.00 (1.08)	2.81 (1.36)	3.00 (1.55)	2.83 (1.72)
Take drugs	1.11 (0.51)	1.27 (0.70)	1.13 (0.47)	1.31 (0.86)	1.00 (0.00)	1.50 (1.23)	1.00 (0.00)
Take sedatives	1.21 (0.52)	1.80 (0.94)	1.23 (0.58)	1.54 (0.97)	1.37 (0.63)	1.33 (0.52)	1.00 (0.00)
Take Medication	1.24 (0.85)	2.93 (1.87)	1.21 (0.77)	2.00 (1.96)	1.63 (1.18)	2.17 (2.04)	1.33 (0.82)
Smoke more	1.26 (0.84)	1.40 (0.83)	1.38 (1.02)	1.38 (0.96)	1.04 (0.19)	1.83 (1.33)	1.00 (0.00)
Talk to intimate partner	3.50 (1.24)	3.80 (1.47)	3.54 (1.14)	3.00 (1.16)	3.74 (1.29)	2.67 (1.37)	4.83 (1.33)
Talk to friends	3.12 (1.01)	2.27 (0.88)	3.03 (1.22)	2.92 (0.76)	3.11 (0.75)	1.83 (0.75)	3.33 (1.03)
Participate in social groups	1.87 (0.98)	1.80 (0.78)	1.87 (0.86)	1.85 (1.14)	1.96 (1.02)	1.17 (0.41)	2.00 (1.10)
Try to be alone	2.89 (1.24)	3.87 (1.06)	2.87 (1.17)	2.69 (1.11)	3.19 (1.36)	4.17 (0.75)	3.33 (1.63)
Engage in Religious activity	2.13 (1.25)	2.40 (1.50)	2.15 (1.23)	2.38 (1.61)	2.15 (1.23)	2.00 (0.89)	2.17 (1.84)
Seek professional help	1.84 (0.99)	3.00 (1.36)	1.87 (1.01)	2.62 (1.50)	1.96 (1.16)	2.33 (1.03)	1.83 (0.98)
Eat more	2.54 (1.24)	3.13 (1.06)	2.33 (1.11)	2.77 (0.93)	3.22 (1.37)	2.17 (0.98)	2.17 (1.33)
Eat less	2.05 (0.91)	2.67 (1.05)	1.92 (0.93)	2.38 (0.77)	2.30 (0.95)	2.50 (1.05)	2.17 (1.33)
Exercise more	2.21 (1.01)	2.80 (1.27)	2.31 (1.17)	2.23 (0.83)	2.48 (1.09)	1.67 (0.82)	2.33 (1.03)

*Note*: Listwise. Values represent mean scores and standard deviations in parentheses. The coping mechanism scale is 1 (Never) to 6 (Always). One = DFE with only one primary role (image/audio/video/detective); Two = combination of two primary roles (e.g., image + video, image + detective, etc.); Three = combination of three primary roles; Four = All four primary roles; Others = digital forensic examiners who did not report one of the primary roles but a general field (e.g., mobile forensics).

There was a marginal difference for the coping mechanism, being alone (*F*(3, 84) = 2.34, *p* = 0.08, ω^2^ = 0.04); Individuals with all four primary roles were more likely to try and be alone as a coping mechanism compared with individuals with one (Bonferroni = 0.13; Gabriel = 0.09) or two primary roles (Bonferroni = 0.09; Gabriel = 0.08); see Table 5.

For PTSD, there were significant mean differences in that individuals meeting the diagnostic criteria for PTSD were more likely to use the following coping mechanisms: “Try to Forget,” *t*(89) = −2.32, *p* < 0.05, *r* = 0.24; “Sleep more than usual,” *t*(89) = −2.46, *p* < 0.01, *r* = 0.25; “Take sedatives,” *t*(15.76) = −2.36, *p* < 0.001, *r* = 0.51; “Take medication,” *t*(15.15) = −3.45, *p* < 0.001, *r* = 0.66; “Try to be alone,” *t*(89) = −2.84, *p* < 0.01, *r* = 0.29; “Seek professional counseling,” *t*(89) = −3.86, *p* < 0.001, *r* = 0.38; and “Exercise more than usual,” *t*(89) = −1.98, *p* = 0.05, *r* = 0.21. There was a marginal difference in that those individuals with PTSD were more likely to “Eat less than usual” as a coping mechanism, *t*(89) = −1.74, *p* < 0.10, *r* = 0.24. Finally, individuals without PTSD were more likely to report “Talking to Friends” as a coping mechanism compared with individuals with PTSD, *t*(89) = 3.05, *p* < 0.01, *r* = 0.31.

### 5.5 Social support

As per Table 2, image analysts were significantly less likely to be satisfied with their social support network than non‐image analysts. There were no differences between the other primary duties. One‐way ANOVA revealed statistically significant mean differences in satisfaction with social support for the number of primary roles reported, *F*(3, 84) = 4.97, *p* < 0.01. ω^2^ = 0.12. Individuals with all four duties reported lower satisfaction with social support compared with individuals with one duty (Bonferroni = 0.002; Gabriel = <0.001) or three roles (Bonferroni = 0.02; Gabriel = <0.01). In addition, there was a significant mean difference in social support satisfaction for PTSD, *t*(92) = 4.31, *p* < 0.001, *r* = 0.41, in that individuals with PTSD scored lower on social support (See Table 3).

### 5.6 Mental health attitudes

As per Table 6, image forensic examiners were significantly more likely to report the following mental health attitudes compared with non‐image analysts: “I don't know where to get help,” “It would be difficult to get time off from work,” and “It is difficult to talk about things I have seen.” There was also a marginally significant correlation in that image analysts were more likely to report that “It is difficult to get an appointment.” Audio forensic analysts were more likely to report that “It is difficult to get an appointment” compared with non‐audio analysts. There was also a marginally significant correlation for “It is difficult to talk about things.” Finally, a marginally significant correlation existed for video forensic examiners reporting, “It is difficult to get an appointment.” In addition, there was a marginal correlation in that non‐video analysts were more likely to state that “It would harm my career” compared with video analysts.

**TABLE 6:** Zero‐order correlation between MHAT and primary duties/DFE vs. DFE + Det.

Mental health advisory stigma	Image	Audio	Video	DFE vs dual	DFE Years^a^
I don't trust mental health professionals	−0.09	−0.03	−0.14	0.06	0.10
Mental health services aren't available	0.05	0.05	0.05	−0.03	−0.12
I don't know where to get help	0.23*	0.07	0.13	−0.02	0.02
It is difficult to get an appointment	0.19^†^	0.21*	0.18^†^	−0.00	−0.16
There would be difficulty getting time off work for treatment	0.25*	0.15	0.18	−0.01	−0.06
It is too difficult to get to the location where the mental health specialist is	0.03	0.03	−0.03	0.04	−0.14
It would be embarrassing	0.08	−0.02	−0.08	0.11	0.07
It would harm my career	0.01	−0.04	−0.18^†^	0.05	−0.01
Members of my unit/department might have less confidence in me	0.08	−0.03	−0.11	0.09	0.02
My superiors might treat me differently	0.06	−0.05	−0.16	0.08	−0.02
My superior would blame me for the problem	0.05	−0.13	−0.04	0.11	−0.05
I would be seen as weak	0.07	−0.03	−0.10	0.08	0.05
It might affect my promotion	0.07	−0.08	−0.15	0.16	−0.01
My superiors discourage the use of mental health services	−0.06	−0.16	0.00	0.02	−0.06
It is difficult to talk about the things I have seen	0.38***	0.19^†^	0.03	0.03	−0.01

*Note*: Listwise *N* = 90; DFE vs. Dual = DFE‐only vs. DFE + Detective, DFE Years = years spent as a DFE; ^†^ *p* < 0.10, *p < 0.05, **p < 0.01, ***p < 0.001.

^a^ *N* = 88.

There were no significant correlations for individuals working as a digital forensic examiner vs. a digital forensic examiner and a detective. Additionally, there were no significant differences in the number of years an individual worked as a digital forensic examiner.

Based on the number of primary roles, there were no significant mean differences in attitudes toward mental health stigmas (see Table 7). However, there were mean differences for 9 of the 15 mental health stigmas when comparing individuals with and without PTSD. As presented in Table 7, individuals with PTSD reported significantly higher mean scores for the following mental health stigmas: “It is difficult to get an appointment,” “I will be seen as weak,” “It will affect my promotion,” “Superiors discourage seeking mental health services,” and “It is difficult to talk about the things I have seen.” There were marginally significant differences in that individuals with PTSD reported higher mean scores for: “Mental health services are not available,” “I don't know where to get help,” “Members of my unit might have less confidence in me,” and “My superiors will blame me for the problem.”

**TABLE 7:** Mean scores on mental health attitude (MHAT) stigma based on number of primary roles reported and PTSD (no/yes).

MHAT	PTSD			Roles				
	No	Yes	Comparison statistics	One	Two	Three	Four	Others
*n*	76	14		40	13	26	6	5
Don't trust mental health professionals	2.25 (1.07)	2.29 (0.83)	*ns*	2.30 (0.94)	2.00 (1.08)	2.19 (1.10)	2.33 (1.21)	2.80 (1.30)
Mental health services aren't available	1.71 (0.80)	2.14 (0.77)	*t*(88) = −1.87, *p* = 0.06, *r* = 0.20	1.83 (0.87)	1.85 (0.56)	1.69 (0.79)	2.00 (1.10)	1.40 (0.55)
Don't know where to get help	1.79 (0.85)	2.29 (1.07)	t(88) = −1.92, *p* = 0.06, *r* = 0.20	1.75 (0.74)	2.00 (1.00)	1.85 (1.01)	2.50 (1.38)	1.80 (0.45)
Difficult to get an appointment	2.38 (1.13)	3.50 (1.16)	*t*(88) = −3.39, *p* < 0.001, *r* = 0.34	2.40 (1.15)	2.31 (1.11)	2.81 (1.33)	3.33 (1.21)	2.20 (0.84)
Difficulty getting time off work for treatment	2.14 (1.21)	2.71 (1.33)	*ns*	2.00 (1.11)	2.38 (1.33)	2.58 (1.36)	2.50 (1.52)	1.60 (0.55)
Too difficult to get to the location where the mental health specialist is	1.87 (0.96)	2.14 (0.95)	*ns*	1.98 (1.03)	1.62 (0.77)	2.12 (1.03)	1.50 (0.55)	1.60 (0.55)
Would be embarrassing	2.37 (1.27)	2.86 (1.23)	*ns*	2.38 (1.15)	2.23 (1.36)	2.62 (1.50)	2.33 (1.21)	2.80 (1.10)
Would harm my career	2.22 (1.22)	2.57 (1.16)	*ns*	2.28 (1.06)	2.08 (1.04)	2.38 (1.47)	1.83 (0.98)	2.80 (1.64)
Members of my unit/department might have less confidence in me	2.24 (1.23)	2.93 (1.33)	*t*(88) = −1.91, *p* = 0.06, *r* = 0.20	2.25 (1.08)	2.23 (1.36)	2.46 (1.39)	2.33 (1.75)	2.80 (1.48)
Superiors might treat me differently	2.33 (1.19)	2.86 (1.46)	*ns*	2.35 (1.08)	2.31 (1.38)	2.46 (1.30)	2.33 (1.75)	3.00 (1.58)
Superiors would blame me for the problem	1.86 (1.04)	2.43 (1.34)	*t*(88) = −1.81, *p* = 0.07, *r* = 0.19	1.88 (0.94)	2.08 (1.26)	1.88 (1.11)	2.17 (1.60)	2.20 (1.64)
Would be seen as weak	2.32 (1.29)	3.21 (1.53)	*t*(88) = −2.33, *p* < 0.05, *r* = 0.24	2.33 (1.16)	2.23 (1.54)	2.50 (1.39)	2.83 (1.72)	3.40 (1.82)
Affect my promotion	2.11 (1.21)	2.93 (1.33)	*t*(88) = −2.31, *p* < 0.05, *r* = 0.24	2.17 (1.15)	1.92 (1.12)	2.19 (1.23)	2.83 (1.72)	3.00 (1.87)
Superiors discourage the use of mental health services	1.59 (0.82)	2.14 (1.29)	*t*(14.98) = −1.54, *p* < 0.05, *r* = 0.37	1.70 (0.82)	1.77 (1.24)	1.58 (0.95)	1.50 (0.84)	2.00 (1.00)
Difficult to talk about the things I have seen	2.64 (1.24)	4.21 (0.89)	*t*(23.38) = −5.65, *p* < 0.001, *r* = 0.76	2.55 (1.22)	2.92 (1.55)	3.31 (1.29)	3.50 (1.05)	2.60 (1.52)

*Note*: Values represent mean scores with standard deviation in parentheses. The MHAT scale is 1 (Strongly Disagree) to 5 (Strongly Agree); One = DFE with only one primary role (image/audio/video/detective); Two = combination of two primary roles (e.g., image + video, image + detective, etc.); Three = combination of three primary roles; Four = all four primary roles; Others = digital forensic examiners who did not report one of the primary roles but a general field (e.g., mobile forensics).

When assessing confidence levels in therapy, there were no significant correlations for the different primary duties. In addition, there were no significant mean differences based on the number of primary roles (see Table 8). However, there were significant differences in confidence levels for therapy between individuals with and without PTSD. As given in Table 8, individuals with PTSD reported significantly higher confidence scores that by the end of therapy, they: “Think symptoms would improve” and “Feel that their symptoms would be reduced.” However, there was a marginal difference in confidence levels that individuals with PTSD “really feel that symptoms would improve” by the end of therapy.

**TABLE 8:** Mean differences in therapy improving symptoms based on number of roles and PTSD (no/yes).

CEQ	PTSD			Roles				
	No	Yes	Comparison statistics	One	Two	Three	Four	Others
*n*	78	16		41	13	28	6	6
By the end of therapy, how much improvement in your symptoms do you think would occur	41.99 (26.34)	63.75 (21.63)	*t*(92) = −3.09, *p* < 0.01, *r* = 0.31	45.39 (28.00)	53.77 (24.27)	41.43 (24.62)	54.33 (35.81)	41.50 (26.86)
At this point, how much do you really feel that therapy will help you reduce your symptoms?	38.56 (27.90)	60.50 (29.87)	*t*(92) = −2.83, *p* < 0.01, *r* = 0.28	39.59 (29.17)	54.92 (32.77)	42.93 (28.11)	43.00 (31.43)	29.83 (25.30)
By the end of therapy, how much improvement in your symptoms do you really feel would occur?	38.69 (28.29)	53.00 (25.07)	*t*(92) = −1.88, *p* = 0.06, *r* = 0.19	39.63 (29.83)	49.62 (28.84)	41.25 (25.42)	43.50 (31.65)	30.00 (28.15)

*Note*: Values represent mean scores and standard deviation in parentheses. The CEQ scale is 0–100%. One = DFE with only one primary role (image/audio/video/detective); Two = combination of two primary roles (e.g., image + video, image + detective, etc.); Three = combination of three primary roles; Four = all four primary roles; Others = digital forensic examiners who did not report one of the primary roles but a general field (e.g., mobile forensics).

Finally, a Chi‐square statistical analysis examined attitudes toward counseling based on primary duties (e.g., image vs. non‐image analyst). Image analysts were more likely to “know someone who has sought counseling,” χ^2^(1) = 4.89, *p* = 0.03, Cramer's *V* = 0.23, “believe that counseling should be required for digital forensic examiners,” χ^2^(2) = 7.68, *p* = 0.02, Cramer's *V* = 0.29, and “think that they need counseling/treatment for work‐related stress,” (χ^2^(2) = 6.21, *p* = 0.05, Cramer's *V* = 0.26). There was a marginal relationship in that video forensic examiners were reported “counseling/treatment as a requirement for their job,” χ^2^(1) = 2.98, *p* = 0.08, Cramer's *V* = 0.18 and “believe that counseling should be required for digital forensic examiners,” χ^2^(2) = 4.71, *p* = 0.10, Cramer's *V* = 0.12. There were no significant associations for audio forensic examiners. Finally, there was a marginal association for individuals with dual roles (digital forensic examiners and detectives) in that they were more likely to seek counseling as compared with the individuals working as digital forensic examiners‐only, χ^2^(1) = 3.03, *p* = 0.08, Cramer's *V* = 0.18.

2 × 4 or 3 × 4 Fisher–Freeman–Halton exact tests (FFHET) determined significant associations based on the number of primary roles and attitudes toward counseling. There was a significant association in that individuals working two roles were more likely to “personally seek counseling” (*p* < 0.01, Cramer's *V* = 0.41). In addition, individuals working two roles were more likely to have sought counseling due to work‐related stress compared with individuals working three roles (*p* < 0.01, Cramer's *V* = 0.41). There were marginal associations for “would you attend counseling if offered but not required” (*p* < 0.10, Cramer's *V* = 0.26), in that individuals working two roles were more likely to be “indifferent” and individuals working three roles were more likely to say “no.” In addition, individuals working all four primary roles were more likely to say “yes” compared with individuals working two roles for “Do you believe counseling should be required” (*p* = 0.10, Cramer's *V* = 0.25).

Individuals meeting the diagnostic criteria for PTSD were more likely to say “yes” to: “have you ever sought counseling/treatment as a result of your work,” χ^2^(1) = 19.36, *p* < 0.001, Cramer's *V* = 0.46; “would you attend counseling or treatment sessions if it were offered but not required,” χ^2^(2) = 15.38, *p* < 0.001, Cramer's *V* = 0.41; “do you think you need counseling or treatment as a result of work‐related stress,” χ^2^(2) = 25.15, *p* < 0.001, Cramer's *V* = 0.52; and “do you think counseling would help improve your productivity at work,” χ^2^(2) = 17.21, *p* < 0.001, Cramer's *V* = 0.43. Finally, there was a marginally significant relationship with “if they believe counseling for digital forensic examiners should be required,” χ^2^(2) = 5.28, *p* = 0.07, Cramer's *V* = 0.24.

## 6 DISCUSSION

Several studies suggest digital forensic examiners experience increasing psychological distress due to exposure to disturbing media [4, 17, 18, 26], especially child sexual abuse material (CSAM; [10, 17]). However, the literature lacks a full BHNAS [13] of digital forensic examiners, which includes barriers to mental health services and attitudes toward counseling [17, 22]. Thus, the goal of this study was to conduct a BHNAS, which addressed the psychological well‐being, mental health barriers, and attitudes toward counseling for DFE and individuals with dual roles (digital forensic examiners and detectives) based on their exposure to image, audio, or video evidence.

We found that digital forensic examiners reported various psychological symptoms consistent with previous studies [4, 10, 17]. Anhedonia and hypersensitivity were consistent symptoms across audio, image, and video forensic analysts, followed by depression, fatigue, and changes in eating habits (for two out of the three roles). Image forensic examiners reported the most psychological distress symptoms, followed by video and then audio forensic examiners. In the current study, 32% (*n* = 30) of participants were not assigned to a specialized unit. Of the 64 participants assigned to a specialized unit, 34% (*n* = 22) were assigned to a child sexual exploitation unit, whereas 45% (*n* = 29) reported being assigned to a “digital forensics unit” in general rather than a specific task force (e.g., ICAC) or criminal unit (e.g., homicide, major crimes).

Although it is difficult to determine why image analysts reported experiencing more psychological distress than video and audio examiners, it may be related to the large quantity and variety of traumatic events (e.g., homicide, child sexual exploitation and abuse, and active shooters) they are exposed to. For instance, the National Center for Missing and Exploited Children's CyberTipline received more than 54.8 million images (41% were unique) and 49.5 million videos (23% were unique) related to child sexual exploitation and abuse in 2023 [60]. In addition, image analysts are exposed to “snapshots in time,” and this lack of context increases psychological discomfort due to the image's ambiguity, leaving the analysts to cognitively process the missing narrative [61]. Puccetti et al. [62] suggest that this cognitive processing of ambiguous information tends to be negative, which leads to adverse effects. Image analysts are also responsible for reviewing and categorizing (i.e., grading) CSAM imagery based on legal definitions to determine the severity of charges [10, 38, 63]. However, some investigators perceive CSAM videos as more distressing than images because they are exposed to the abuse from beginning to end [17].

Overall, units specifically investigating child sexual exploitation and abuse (CSEA) cases are taking steps to reduce psychological distress from exposure to CSAM imagery. Some units include wellness policies that recommend the examiners mute the videos when they are watching them, or they do not watch the videos when listening to the audio [17, 24, 37]. In addition, it is recommended that examiners view CSAM images as thumbnails [17]. Overall, it is essential that digital forensic examiners have control of how and when they view the CSEA material to increase resilience and reduce psychological distress [5, 64].

Contrary to Seigfried‐Spellar [10], we found no statistically significant differences in psychological well‐being between digital forensic examiners and individuals working dual roles as digital forensic examiners and detectives. In addition, individuals with digital forensics‐only roles were more likely to meet the PTSD diagnostic criteria as compared with the individuals with dual roles (digital forensic examiners and detectives)– again, which contrasts findings from Seigfried‐Spellar [10]. Recent research emphasizes the importance of case‐related feedback in reducing work‐related stress, especially if employees are dealing with child exploitation cases [5]. If digital forensic examiners know the outcome of the case (e.g., exonerating a suspect, identifying a new victim, or an offender pleads guilty) based on their analysis of the digital forensic artifacts, this feedback validates the importance of their work, thereby improving mental health and job satisfaction [4, 24, 38]. Thus, individuals assigned dual duties as detectives and digital forensic examiners may be more likely to know the case's outcome, which may be a protective factor for poor psychological well‐being. However, it should be noted that digital forensic examiners with all four primary roles (image, audio, video, and detective) scored higher on PTSD as compared with the individuals having one (image/audio/video). This finding is likely due to the high caseload and volume of digital forensic artifacts, which increase exposure to various disturbing media [5, 17].

As per the current study, most digital forensic examiners reported being somewhat or extremely satisfied with their job, consistent with previous studies [9, 29]. However, job satisfaction may be influenced by a wide range of variables, including resource shortages and organizational culture and support, making it a complex variable when trying to assess wellness and resiliency. For instance, technical skills are important for digital forensic examiners in terms of job satisfaction [22], and budget restrictions may impact training opportunities or the ability to purchase new forensic tools, thereby reducing satisfaction. In addition, digital forensic examiners engage in different activities to increase job satisfaction and cope with work‐related stress. Consistent with previous studies [10, 24], individuals with dual roles (digital forensic examiners and detectives) tend to cope by using distraction/suppression and changing eating habits (e.g., eating less). In addition to distraction/suppression, image and video analysts relied on medications, such as antianxiety pills, as a coping strategy. As per other studies, “talking with others” [10, 17, 18] and “engaging in religious activities” [10, 18, 19] were also common coping mechanisms for digital forensic examiners, but in the current study, they were not significant factors based on roles and primary duties.

When comparing the coping strategies used based on the number of primary roles, individuals with four primary roles were less likely to talk with others than individuals with one primary role. They also used escapism as a coping mechanism. These findings suggest that participants engaged in various roles may experience more work‐related stress and emotional burdens due to the diverse nature of their responsibilities as digital forensic examiners. Moreover, individuals with multiple roles may feel an increased sense of isolation because others may not understand their experiences on an emotional level. Digital forensic examiners face difficulty in discussing their experiences with friends, family, or professionals because of a concern that sharing the details of distressing material would traumatize them [5, 17]. However, we did find that individuals who met the PTSD diagnostic criteria were more likely to seek professional help, although they also reported using substances and escapism as coping strategies.

The results also indicate that digital forensic examiners, especially when dealing with images or having multiple primary roles, were less likely to be satisfied with their social support network (i.e., social support external to work). Moreover, individuals meeting the PTSD diagnostic criteria were less likely to be happy with their social support network. Research suggests that digital forensic examiners prefer talking with others with the same skillset and experience, as their peers are more likely to understand their work‐related stress and burdens [17, 24]. As previously mentioned, digital forensic examiners are afraid to talk about work‐related stress with their friends and family. Thus, their dissatisfaction with their social support network may be confounded by their fear of speaking with individuals external to work. Having close relationships supporting their work may help to reduce PTSD symptoms [19, 65] and improve mental health [66], as the absence of a social support network may elevate PTSD symptoms and increase the risk of experiencing negative situations outside the workplace [22].

Digital forensic examiners and detectives would likely benefit from considering mental health services to reduce psychological distress and increase resiliency due to the nature of their jobs. Compared with 9.3% of participants from Seigfried‐Spellar's study [10], we found that over one‐third (36.2%) of the individuals reported seeking counseling due to their work‐related stress. However, there was only a slight increase in the number of examiners who reported that counseling/treatment was required as a part of their job (19% vs. 16%; [10]). In addition, there was little change in the number of participants (50%) who believed that counseling should be required (52%; [10]). In the current study, individuals with dual roles were more likely to seek counseling than individuals working only as digital forensic examiners. Moreover, individuals with PTSD symptoms were more likely to have sought counseling because of work‐related stress; they also reported believing that counseling would improve their productivity at work.

The continued hesitance to seek or require counseling may be related to the stigma associated with or barriers toward seeking mental health support. Based on primary duties, the results suggest different challenges related to mental health services, predominantly related to accessing mental health resources and comfort with seeking support. Image forensic examiners were more likely to report “not getting time off to receive mental health services” or “being unaware of where to get help.” In addition, image and audio analysts reported that it was “difficult to talk about the things they have seen.” All three primary roles (image, audio, and video forensic examiners) reported that it was “difficult to get an appointment.” Finally, the results indicated no significant differences in digital forensic examiners‐only and dual roles as detectives toward mental health services attitudes.

Overall, these findings align with several of the policy recommendations made by Mitchell et al. [37], including increasing awareness of and accessibility to wellness programs and strategies, providing flexible work hours, preparing personnel for the potential work‐related stress and mental health impact, supplementing employee assistance programs, and developing resources for agencies without supportive unions. In the current study, the examiners' barriers to seeking mental health support were more related to access and availability rather than fear associated with agency culture, such as being seen as weak [37] or being reassigned or removed from their current post [38]. Overall, the lack of knowledge and wellness resources (e.g., difficulty getting an appointment, where to get help), coupled with difficulty getting time off from work (stress leaves; [5]), possibly related to increasing caseload and backlog, appear to be critical barriers for digital forensic examiners seeking mental health support.

However, those digital forensic examiners that met the diagnostic criteria for PTSD reported 9 out of 15 mental health stigmas, many of which included fear associated with agency culture (e.g., “superiors would blame me for the problem;” “be seen as weak;” “affect my promotion;” “members of my department would have less confidence in me;” “superiors discourage the use of mental health services”). This finding is consistent with the study conducted by Drew and Martin [67], who found that police officers with PTSD symptoms were more likely to endorse stigmas associated with seeking mental health services and had negative attitudes about recovery. Overall, digital forensic examiners believe they will encounter barriers to seeking mental health services, especially those experiencing PTSD symptoms.

Within the criminal justice literature, there is a debate as to whether mental health services should be voluntary or mandatory. Mandated counseling, especially after critical incidents, not only introduces law enforcement personnel to mental health resources [68] but also encourages continued engagement when compared with voluntary services [68, 69]. Law enforcement personnel were also more likely to report concerns regarding perceived value and trust when the services were voluntary compared with mandatory [68]. In addition, mandatory mental health services may reduce the stigma associated with help‐seeking behavior [10, 37, 38]. However, Papazoglou and Tuttle [70] state that mandatory referrals for mental health services may be viewed as a punishment or as questioning one's professional competence, which may lead to further resistance.

Another problem associated with mandatory mental health resources is the lack of specialized clinicians familiar with criminal justice occupations. In general, the Commonwealth Fund reports that there is an acute behavioral health workforce shortage in the US [71]. Additionally, there is a critical and widespread shortage of culturally competent mental health professionals, particularly those equipped to understand and serve the unique contexts of law enforcement and forensic fields [72]. Overall, the research supports the need for accessible, agency‐supported mental health services, noting that mandatory resources could reduce stigma but may be difficult to implement.

### 6.1 Limitations and future research

This study is not without limitations. The sample size was relatively small (*N* = 94), but this is partly because we required the respondents to be *certified* and *currently working as* digital forensic examiners in the US. Similar research studies with larger sample sizes often include *any* personnel exposed to disturbing media (e.g., detectives, investigators, and digital forensic analysts; [5, 24]). Our sample, which focuses on certified digital forensic examiners currently working in the US, may limit the generalizability of policy recommendations when compared with more diverse samples, that include any personnel exposed to disturbing media. In addition, the response rate may be lower as the survey contained sensitive questions regarding their attitude toward mental health services and psychological well‐being. The sensitive nature of the survey may also introduce a social desirability bias; however, the survey was anonymous (i.e., no identifying information was collected) to reduce bias and increase honesty in responses.

More research is needed to understand robust ways to improve agency culture and reduce mental health stigma and barriers toward seeking help for digital forensic examiners exposed to disturbing media. For instance, the stigma associated with help‐seeking behavior may be reduced if agencies make mental health services a mandatory component of their wellness policies [10, 37, 38]. However, requiring mental health services is difficult for smaller agencies with limited resources [37, 38], as well as identifying qualified counselors who understand the demanding job duties of digital forensic examiners [17, 73]. The academic literature continues to suggest that digital forensic examiners exposed to disturbing media experience poor psychological well‐being and barriers toward help‐seeking.

Overall, future research is needed to understand the successful wellness policies and awareness programs that build resilience and reduce job‐related stress for DFE exposed to disturbing media. The wellness programs may include stress management, mental health care, peer and family support, financial guidance, and training on both safety and tactical skills [37]. While our study considered participants' roles and duties within their agencies, we did not collect detailed information on their exposure to different types of content (e.g., suicide, beheading). Thus, including detailed information on the specific types of digital content they examine may help identify higher‐risk groups and inform the design of wellness programs tailored to the needs of specific exposure types. Moreover, the current study only examined the number of years participants worked as a DFE, so future research is needed to understand the cumulative nature of not only exposure‐related distress as a digital forensic examiner but also in cases where the individual is also working as a detective/investigator.

## 7 CONCLUSION

In conclusion, the current study addresses a critical gap by conducting a behavioral health needs analysis of digital and multimedia forensics examiners by examining general psychological well‐being, PTSD, coping mechanisms, social support, and attitudes toward and barriers to seeking mental health services. The authors conclude that based on primary duties, individuals working as image forensic analysts report more psychological distress, followed by video than audio forensic examiners. In addition, individuals working all four duties (image, audio, video, and detective) were more likely to meet the diagnostic criteria for PTSD. The study also highlights that PTSD‐symptomatic individuals believe they will encounter more barriers toward seeking mental health services, including stigmas associated with agency culture. Overall, our findings support the need for mandatory and accessible mental health services as well as a supportive work culture that normalizes help‐seeking behaviors to resolve the challenges associated with poor psychological well‐being and increased stigma for DFE experiencing work‐related stress.

## CONFLICT OF INTEREST STATEMENT

The authors have no conflicts of interest to declare.

## Data Availability

The data that supports the findings of this study are available in the supplementary material of this article.
